# Whole exome sequencing in Chinese mucinous pulmonary adenocarcinoma uncovers specific genetic variations different from lung adenocarcinoma

**DOI:** 10.3389/fonc.2022.1054845

**Published:** 2022-12-15

**Authors:** Chenyue Zhang, Kai Wang, Wenjie Liu, Jiamao Lin, Zhenxiang Li, Hui Wang, Chenglong Zhao, Yanhua Chen, Shuangxiu Wu, Airong Yang, Jiayan Wu, Haiyong Wang

**Affiliations:** ^1^ Department of Integrated Therapy, Fudan University Shanghai Cancer Center, Shanghai Medical College, Shanghai, China; ^2^ Key Laboratory of Epigenetics and Oncology, the Research Center for Preclinical Medicine, Southwest Medical University, Luzhou, China; ^3^ Research Institute, Berry Oncology Corporation, Beijing, China; ^4^ Department of Traditional Chinese medicine, Shandong Cancer Hospital and Institute, Shandong First Medical University and Shandong Academy of Medical Sciences, Jinan, China; ^5^ Department of Radiation Oncology, Shandong Cancer Hospital and Institute, Shandong First Medical University and Shandong Academy of Medical Sciences, Jinan, China; ^6^ Department of Thoracic Surgery, Shandong Provincial Hospital Affiliated to Shandong First Medical University, Jinan, China; ^7^ Department of Pathology, The First Affiliated Hospital of Shandong First Medical University and Shandong Provincial Qianfoshan Hospital, Jinan, China; ^8^ Department of Biological Information, Berry Oncology Corporation, Beijing, China; ^9^ Department of Internal Medicine-Oncology, Shandong Cancer Hospital and Institute, Shandong First Medical University and Shandong Academy of Medical Sciences, Jinan, China

**Keywords:** whole exome sequencing, mucinous pulmonary adenocarcinoma, genomics, immunological features, therapeutics

## Abstract

**Background:**

As a rare subtype of primary lung adenocarcinoma (LUAD), mucinous pulmonary adenocarcinoma (MPA) was considered a distinctive entity with unfavorable outcomes. Therefore, there is a great need for a better understanding of the genomic and immunological landscape of this rare tumor type, which would inform improved therapeutic strategies.

**Methods:**

A total of 96 patients histologically confirmed with MPA were recruited from Shandong Cancer Hospital and Institute (SCH). Single nucleotide variation (SNV), copy number variation (CNV), genomic instability, and immunological landscape insights into 96 MPA patients were identified using WES.

**Results:**

We demonstrated that MPAs had marked different genomic alterations and were more complex in genomic profiles than LUADs. Mutations in Tumor Protein 53 (*TP53*) and CYP7A Promoter-Binding Factor (*CPF)* pathways significantly shortened survival whereas mutations in *Notch* and *Wnt* pathways significantly prolonged survival in MPA. Besides, we demonstrated that mutations in immune-related genes influenced outcomes, with mutations in *TP53*, Ataxia Telangiectasia Mutated *(ATM)*, Polymerase (DNA) Delta 1 (*POLD1)*, and Epidermal Growth Factor Receptor *(EGFR)* correlated with worsened survival.

**Conclusions:**

We not only depicted the genetic and immunologic landscape of Chinese MPA but also reveal its distinction from LUAD in genomic and immune context. Our findings may provide opportunities for therapeutic susceptibility among Chinese MPA patients.

## Introduction

Mucinous pulmonary adenocarcinoma (MPA) is defined by the WHO classification as primary lung adenocarcinoma (LUAD) with tumor cells demonstrating goblet cell or columnar cell morphology with abundant intracytoplasmic mucin (1). As a rare subtype of primary LUAD, it comprises approximately 3%-5% of adenocarcinoma (2). Despite multi-modality aggressive therapies, the overall survival for patients with MPA is dismal (3, 4). Neither platinum-based chemotherapy nor targeted therapy is effective for MPA (5, 6). Given these important differences from LUAD, MPA was considered a distinctive entity with unfavorable outcomes. Therefore, there is a great need for a better understanding of the genomic landscape of mucinous pulmonary adenocarcinoma (MPA), which would inform improved therapeutic strategies.

Nevertheless, much less is known about the genomic alterations of this rare type of tumor, in contrast to the abundance of genomic information about LUAD (7, 8). Recently, several studies have identified mutations of Kirsten Rat Sarcoma *(KRAS)*, B-Raf Proto-Oncogene *(BRAF), Erb-B2 Receptor Tyrosine Kinase 2 (ERBB2)*, and Phosphatidylinositol-4,5-Bisphosphate 3-Kinase Catalytic Subunit Alpha (*PIK3CA)* mutations whereas rare *EGFR* occurred in MPA samples (9). Some gene rearrangements were identified in MPA, including Neuregulin 1 (*NRG1), BRAF, Neurotrophic Receptor Tyrosine Kinase 1 (NTRK1), ALK Receptor Tyrosine Kinase (ALK), Ret Proto-Oncogene (RET)*, and Erb-B2 Receptor Tyrosine Kinase 4 (*ERBB4)* (10). However, due to the limitation of sample size, the detailed characterization of the genomic landscape in MPA, and the exact role of these mutations affecting the survival has remained largely unknown.

In addition, immune checkpoint inhibitors (ICIs) have revolutionized the treatment paradigm in LUAD (11). As the rare subtype of adenocarcinoma, understanding the characterization of immunological features is necessary to boost our understanding and provide underpinnings and rationale for the adoption of ICI in MPA.

In this study, we conduct a comprehensive analysis to identify the genetic variations in 96 MPA patients, including single nucleotide variation (SNV), copy number variation (CNV), genomic instability, and immunological landscape, the largest cohort to date among Chinese population in this rare disease. By integrating multi-platform studies with their clinical information, we reveal the unique genetic and immunological features of MPA, which may translate into therapeutic targets and indicate prognosis. Particularly, we demonstrated that mutational profiles of MPA are distinct from LUAD. These findings further deepen our understanding of MPA and may translate into clinical utility.

## Materials and methods

### Sample collection and genomic DNA extraction

A total of 96 patients histologically confirmed with mucinous pulmonary adenocarcinoma (MPA) were recruited from Shandong Cancer Hospital and Institute (SCH). They were diagnosed with MPA from 14, April 2015 to 19, August 2019, except for one patient who was pathologically confirmed with MPA on 26, December 2013. The last follow-up date was 22, July 2021. All the diagnoses were independently confirmed by two experienced pathologists. All patients in this study provided written informed consent and this study was approved by the Ethics Committee of SCH. It also conforms to the provisions of the Declaration of Helsinki. MPA specimens and attached non-tumor samples were obtained by biopsies. A strict quality inspection was carried out to remove contaminated and insufficient DNA samples. The overall survival (OS) time was defined as the interval between diagnosis and death, or between diagnosis and the last observation point. Clinical pathological data were retrieved from patients’ medical records. Biopsied tumor tissues were fixed with formalin, then embedded in paraffin (FFPE). Corresponding non-tumor samples were set as controls. Genomic DNA was extracted from each FFPE sample using the GeneRead DNA FFPE Kit (Qiagen, #180134, USA) and from the blood sample using the DNA Blood Midi/Mini kit (Qiagen, #51185, USA).

### DNA library construction and whole-exome sequencing

An amount of 4-5μg genomic DNA was firstly enzymatically digested into 200 bp fragments using a 5X WGS Fragmentation Mix kit (Qiagen, #Y9410L, USA) for library construction. T-adapters were added to both ends after repairing and A tailing. For the whole-exome sequencing (WES) library construction, purified DNA was amplified by ligation-mediated PCR. Then, final sequencing libraries were generated using the 96 rxn xGen Exome Research Panel v1.0 (Integrated DNA Technologies, USA), according to the manufacturer’s instructions. Paired-end multiplex samples were sequenced using the Illumina NovaSeq 6000 System (Illumina, USA). The sequencing depth of the tissue sample was 200× per and the white blood cell (WBC) sample sequenced depth was 100× per.

### Sequence data processing and alignment of the MPA cohort

Raw sequencing data were preprocessed by FASTP to trim adaptor sequences (12). Then, clean reads in Fast Q format were aligned to the reference human genome (hg19/GRCh37) by Burrows-Wheeler Aligner (BWA, v0.7.15) (13). SAM tools (14) and Picard (2.12.1) (http://picard.sourceforge.net/) were used to sort mapped BAM files and process PCR duplicates. To compute the sequencing coverage and depth, final BAM files were generated by GATK (Genome Analysis Toolkit 3.8) for local realignment and base quality recalibration (15).

### Public data of LUAD patients

The WES mutation files of 299 LUAD patients were retrieved from a previous study that used the East Asian LUADs cohort for somatic mutational signature and alteration frequency analyses (16). All enrolled samples (N=299) in the LUAD cohort were individuals of East Asian ancestry, and the individuals which were diagnosed as mucinous pulmonary adenocarcinomas were excluded (N=6). Some of Chinese patients’ WES data were taken from a recent study by Beijing Genome Institute (BGI) (17) and others were from patients of Chinese descent from Singapore. All specimens of these patients were reviewed by pathologists.

### Somatic mutation variants detecting and driver gene prediction

Single nucleotide variations (SNVs) and small insertions and deletions (Indels: <50 bp) were identified from clean sequencing data by GATK MuTect2 (v1.1.4) (18) with default parameters. Subsequently, we removed mutations, which were referred to the ENCODE Data Analysis Consortium blacklisted regions (19). We filtered out the SNVs with <20 X depth or 4 X depth of the alternate alleles in tumor or SNVs with <10 X depth in normal or variant reads >1% of normal reads. The ANNOVAR software was used for variants annotation based on multiple databases (20), including variant (HGVS), population frequency (1000 Genomes Project, dbSNP, ExAC), variant functional prediction (PolyPhen-2 and SIFT), and phenotype or disease (OMIM, COSMIC, ClinVar) databases. After annotation, we excluded the SNVs that were annotated as genomicSuperDups and VAF <0.2 or PopFreqMax >0.05 and then screened with VAF (variant allele frequency) ≥ 1% for cancer hotspots which were collected from the patient databases or VAF ≥ 3% for others. The retained mutations were used for further analysis. Tumor mutation burden (TMB) was calculated with the total numbers of non-synonymous SNVs and indel variants per megabase of coding regions. Dominant tumor neoantigens were predicted using OptiType to infer the individual HLA type (21). Significant driver genes were identified by combining MutsigCV and dNdScv, as previously described (22, 23), with a false discovery rate (FDR) cutoff <10%. Genes with significantly different mutation frequencies among the groups were determined based on the gene mutation rates in each cluster using a two-sided Fisher’s exact test with a *P*-value of 0.05.

### Mutational signature analysis

Somatic mutational signatures were *de novo* analyzed from the clean WES data by the “Somatic Signatures” R package (v2.20.0) (24), according to a non-negative matrix factorization (NMF) method. Three highly confident mutational signatures were derived in the MPA cohort. Then, they were compared with the consensus signatures in the COSMIC dataset (https://cancer.sanger.ac.uk/cosmic/), based on the Cosine similarity analysis to nominate each derived signature with the highest COSMIC dataset. To further determine mutational signatures’ distribution and frequencies of each patient, the deconstruct Sigs (v1.9.0) was used as previously described (25). Mutational signatures of each patient were considered to determine MPA subgroups. We inferred patient clusters based on the four mutational signatures’ weights in each patient using “Ward. D2’s method” R package based on maximum distance (16).

### Copy number variation identification

Copy number variations (CNVs) were first identified using the Genome Identification of Significant Targets in Cancer (GISTIC) 2.0 algorithm (26). At the chromosomal arm level, significant amplifications or deletions were screened with FDR (cutoff < 10%) for further analyses. At a focal CNV level, significant amplification was screened with FDR (cutoff < 5%) and G-score (cutoff > 0.3). Significant deletion was screened with FDR (cutoff < 5%) and G-score (cutoff < -0.2) for further analyses. Focal CNV-related gene analysis was performed for each patient based on paired tumor-normal WES data using GATK Depth of Coverage with parameters (–min Base Quality 0 –min Mapping Quality 20 –start 1 –stop 500 –nBins 200 –include Ref NSites –count Type COUNT_FRAGMENTS). Amplified genes were defined by a copy number ratio of tumor vs normal > 4, while deleted genes were defined by a copy number ratio of tumor vs normal < 0.5. Then, focal CNV-related genes were filtered according to the COSMIC cancer gene census database (https://cancer.sanger.ac.uk/cosmic/) to obtain a cancer-related focal CNV gene list. Genes with significantly different CNV frequencies among the different groups were determined based on the gene alteration rates in each cluster using a two-sided Fisher’s exact test with a *P*-value of 0.05.

### Pathway and functional enrichment analysis

Somatic mutation and focal CNV-related genes enriched biological functions and involved pathways were analyzed using the cluster Profiler (R package), based on the Gene Ontology (GO) database (http://geneontology.org/) and Kyoto Encyclopedia of Genes and Genomes (KEGG) databases (https://www.kegg.jp/kegg/kegg1.html). Somatic mutations and focal CNV-related genes were also evaluated by canonical oncogenic signaling and DNA damage repair (DDR) pathways mapping, according to the templates from the TCGA PanCancer Atlas project (27, 28). A pathway was considered “altered” when it contained equal to or more than 1 gene altered in a specimen. The number of oncogenic signaling or DDR pathway alterations of each specimen was also calculated. A comparison of each specific pathway alteration frequency among the different groups was performed using Fisher’s exact test with a *P*-value of 0.05.

### Tumor heterogeneity and genome instability analysis

To investigate intratumor heterogeneity (ITH), mutant allele tumor heterogeneity (MATH) values for each tumor sample were calculated from the median absolute deviation (MAD) and the median of its mutant-allele fractions at tumor-specific mutated loci: MATH = 100 × MAD/median. Detailed information could be found in the **Supplementary Materials and Methods**.

### The evaluation of PD-L1 expression, CD8+ T cell infiltration

The detailed procedures were demonstrated in **Supplementary Materials and Methods**.

### Statistical analysis

The R Foundation for Statistics Computing Package (R package, version 4.0.3) was used to perform the statistical analyses. The Fisher exact test (for categorical variables) and the Wilcoxon rank-sum test (continuous variables) were used to analyze the relationship between the two groups. The Kaplan-Meier method was used to estimate effects on OS times based on Log Rank tests. A *p*-value < 0.05 was defined as statistically significant. Hazard ratios of multiple factors on OS time were obtained from the Cox proportional hazards model.

## Results

### Patient characteristics

After confirmation by pathologists, a total of 96 mucinous pulmonary adenocarcinomas (MPA) patients were enrolled in MPA cohort for molecular mutation detection in this study. Given that MPA is a special pathological type of LUAD, we also included 299 LUAD cases (16) as the LUAD cohort (MPAs were excluded) for genomic variant identification and comparative analysis. The detailed clinical information of both MPA and LUAD patients is shown in **Table 1**. In the MPA cohort, a total of 19 patients were above the age of 65, whose number was much smaller than those below 65. There were 56 females in the MPA cohort, accounting for 58.3%, leaving 40 males making up 41.7%. The majority (64/96) of MPA cases had no smoking history, occupying 66.7%. The stage distribution of the MPA cohort was also analyzed. A total of 74 cases were at stage I, accounting for 77.1% whereas only 4 cases were at stage II (4.2%) and 14 cases at stage III (14.6%). The clinical characteristics of LUAD were also depicted in **Table 1**. In the LUAD cohort, a total of 133 patients were above the age of 65. A significant difference was detected between the MPA and LUAD cohort in the age distribution (*P*=1.18E-05) and clinical stage distribution (*P*=3.32E-08).

**Table 1:** The clinicopathological information of mucin-producing adenocarcinoma of the lung (MPA) and lung adenocarcinoma (LUAD).

	MPA	LUAD	Total	*P* value
Age				
>65	19	133	152	1.18E-05
<=65	77	166	243	
Gender				
Female	56	147	203	0.1281
Male	40	152	192	
Smoking				
Yes	32	107	139	0.6256
No	64	188	252	
NA	0	4	4	
Stage				
I	74	133	207	3.32E-08
II	4	55	59	
III	14	90	104	
IV	1	19	20	
NA	3	2	5	

### Genomic landscape of MPA patients

**Figure 1:**
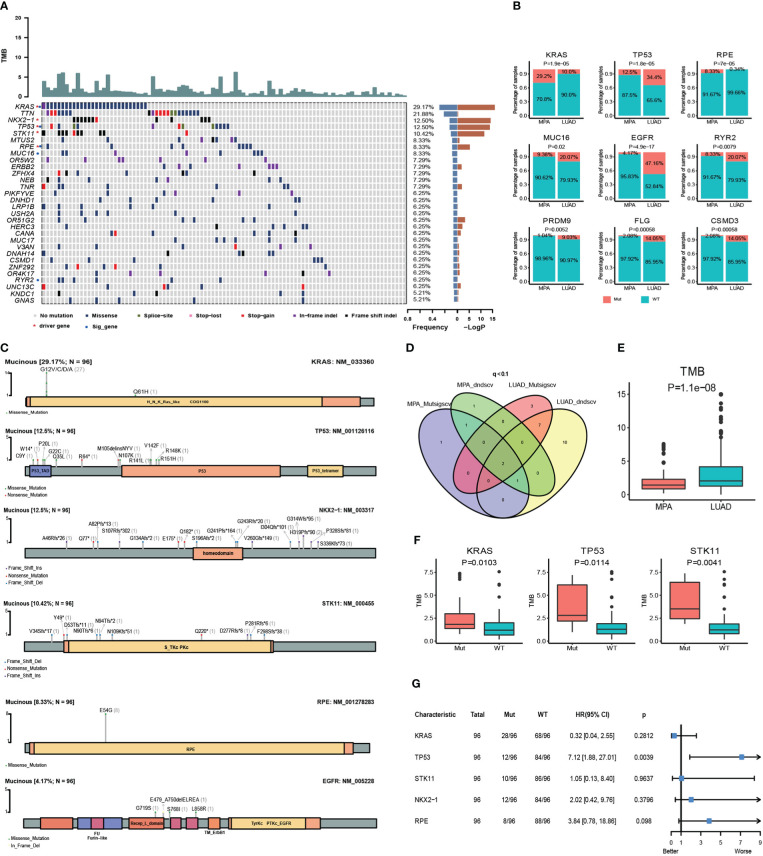
Somatic mutations and clinical association in MPA. **(A)**, Recurrently mutated genes and mutant frequencies of top 30 in lung mucinous adenocarcinoma. The blue in the histogram on the right shows the mutation frequency of these 30 genes, and the red is the -log *P*-value when predicting whether the gene is a driver gene. There are red asterisks (driver genes predicted by the software) and blue circles (genes with a significant difference in mutation frequency between lung mucinous adenocarcinoma and lung adenocarcinoma) on the right of the gene name on the left. **(B)**, Compared with lung adenocarcinoma, lung mucinous adenocarcinoma has a significantly different mutation frequency. In the histogram, red indicates the frequency of mutation, and blue indicates the frequency of no mutation. **(C)**, Lollipop diagram of five driver genes and EGFR mutations in MPA. The specific mutation type of each gene and the number of mutations of each mutation type are marked on the map. **(D)**, Venn diagram of the number of MPA and LUAD driver genes predicted by Mutsigscv and dndscv software. **(E)**, TMB comparison between MPA and LUAD. **(F)**, Comparison of TMB between mutant and wild-type in five driver genes of MPA. Red is the mutant type, and blue is the wild type. **(G)**, Survival analysis of five driver genes in MPA.

Whole-exome sequencing (WES) was performed on tumor samples with a mean depth of 200× and a mean depth of 100× for peripheral blood. The recurrently mutated genes and the top 30 genes with the most mutant frequencies in the MPA cohort was shown in **Figure 1A**. *KRAS* was the most frequently mutated gene in the MPA cohort. The other top frequently mutated genes were Titin (*TTN), NK2 Homeobox 1 (NKX2-1), TP53, Serine/Threonine Kinase 11 (STK11), Microtubule Associated Scaffold Protein 2 (MTUS2), Ribulose-5-Phosphate-3-Epimerase (RPE), Mucin 16 (MUC16), Olfactory Receptor Family 5 Subfamily W Member 2 (OR5W2)*, and *ERBB2*. A total of 29.1% (28/96) patients harbored different form mutations in *KRAS*. These mutations were demonstrated in the forms of missense, splice-site, stop-lost, stop-gain, in-frame indel, and frame-shift indel. We also compared genes with high-frequency mutations in LUAD. The results showed that the top ten mutated genes with high frequency were Epidermal Growth Factor Receptor (*EGFR), TP53, TTN, Ryanodine Receptor 2 (RYR2), Usher Syndrome 2A (USH2A), MUC16, Zinc Finger Homeobox 4 (ZFHX4), CUB And Sushi Multiple Domains 3 (CSMD3), Filaggrin (FLG)*, and LDL Receptor Related Protein 1B (*LRP1B)* in LUAD (**Figure S1A**). The MPA and LUAD cohorts shared the same mutations in *TTN, TP53, MUC16, ZFHX4, Low Density Lipoprotein Receptor-Related Protein 1B (LRP1B), USH2A, Mucin 17 (MUC17), CUB And Sushi Multiple Domains 1 (CSMD1)*, and *RYR2*. The coexistence of several genetic mutations could be observed both in the MPA and LUAD cohorts. Compared with LUAD, MPA had a significantly different mutation frequency in *KRAS, TP53, RPE, MUC16, EGFR, RYR2, PR/SET Domain 9 (PRDM9), FLG*, and CUB And Sushi Multiple Domains 3 (*CSMD3)* (*P<0.05* for all) (**Figure 1B**).

To characterize the potential driver events for MPA, we analyzed *TP53*, *NKX2-1, STK11, RPE*, and *EGFR* mutations among 96 MPA patients. A lollipop diagram of five driver gene mutations in MPA was shown in **Figure 1C**. The number of driver genes between MPA and LUAD were shown in the Venn diagram in **Figure 1D**. The driver genes of MPA and LUAD cohorts were listed in **Table 2**.

**Table 2:** Comparison of driver genes between MPA and LUAD.

MPA		LUAD	
Mutsigscv	dndscv	Mutsigscv	dndscv
KRAS	KRAS	KRAS	KRAS
TP53	TP53	TP53	TP53
NKX2-1	NKX2-1	EGFR	EGFR
STK11	STK11	RBM10	RBM10
RPE	PRR4	RHPN2	RHPN2
		SMAD4	SMAD4
		RB1	RB1
		KEAP1	KEAP1
		PTEN	PTEN
		COPS4	STK11
		STMN1	APC
		DCDC1	KCNA6
			EPRS
			POTEE
			SLC34A2
			ERBB2
			TSHZ3
			FRG1B
			SETD2
			SCLT1

The TMB of LUAD was significantly higher than that of MPA (*P*=1.10E-08) (**Figure 1E**). In MPA, five driver genes showed higher TMB, including *KRAS* (*P*=1.03E-02)*, TP53* (*P*=1.14E-02), and *STK11* (*P*=4.10E-03) (**Figure 1F**). In LUAD cohort, *KRAS* (*P*=1.00E-04)*, TP53* (*P*=3.80E-07), and Doublecortin Domain Containing 1 (*DCDC1)* (*P*=1.31E-02) showing higher TMB, whereas lower in *EGFR* (*P*=1.00E-04) (**Figure S1B**). We further investigated the impact of these driver genes on the overall survival (OS) of MPA. It is observed that the mutation of *TP53* had a significant impact on the prognosis of MPA. Compared with wild-type *TP53*, *TP53*-mutant patients showed significantly worse survival (*P*=3.90E-03, HR, 7.12; 95%CI (1.88-27.01)). However, the mutation of *KRAS, STK11, NKX2-1*, and *RPE* had no significant influence on OS for MPA patients (*P*>0.05) (**Figure 1G**). We also explored the effect of driver genes on survival in the LUAD cohort, which revealed that *EGFR, Kelch Like ECH Associated Protein 1 (KEAP1)*, and Phosphatase And Tensin Homolog (*PTEN)* mutation influenced survival. Mutation in *EGFR* was found to be an independent prognostic factor for better survival (*P*=6.00E-04, HR 95%CI 0.50 (0.33-0.74)). Wild-type *KEAP1* (*P*=1.00E-04, HR, 3.90; 95%CI (2.02-7.49)) and *PTEN* (*P*=2.70E-03, HR, 3.58; 95%CI (1.56-8.21)) were found to be independent factors for worse survival (**Figure S1C**). These specific mutated genes may provide some molecular explanations for the occurrence of MPAs.

### Genomic copy number alterations and statistics of driver genes in hotspot mutation regions

**Figure 2:**
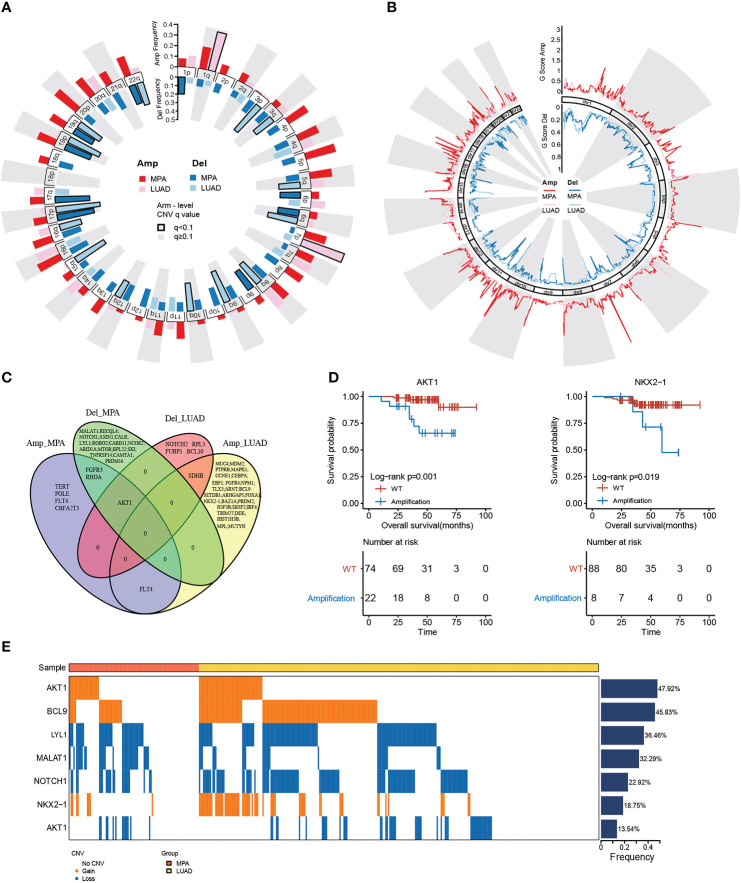
Genomic copy number alterations and statistics of driver genes in hotspot mutation regions. **(A)**, Chromosome arm-level CNV frequencies in MPA and LUAD cohorts. Dark red represents the CNV amplification result of MPA, light red represents the CNV amplification result of LUAD. Dark blue represents the CNV censored result of MPA, and light blue represents the CNV censored result of LUAD. Columns with black borders indicate chromosome arms that have been significantly amplified or censored. **(B)**, Focal-level CNV across chromosomes 1–22, with GISTIC FDR q values on the x-axis. Dark red represents the CNV amplification result of MPA, light red represents the CNV amplification result of LUAD, dark blue represents the CNV censored result of MPA, and light blue represents the CNV censored result of LUAD. **(C)**, Venn diagram of driver gene overlap in the significant copy number region between MPA and LUAD. **(D)**, Survival analysis results of the driver genes above the copy number region in MPA. **(E)**, Differences in CNV of the driver genes shared by MPA and LUAD.

The copy number variations (CNVs) of two cohorts were identified using the Genome Identification of Significant Targets in Cancer (GISTIC) 2.0 algorithm. At the chromosomal level, the MPA cohort showed a higher degree of amplification than the LUAD cohort, while chr 1q and chr 7p were significantly higher in the LUAD cohort (**Figure 2A**). Arm level deletions of the MPA cohort were lower than the LUAD cohort, while chr 1p, chr 6p/q, chr 9p, chr 17p/q, chr 19p/q, chr 22q showed high-level deletions (**Figure 2A**). The focal CNV profiles between the MPA and LUAD cohorts were compared to identify novel focal events (**Figure 2B**). Significantly amplification of Ras Homolog Family Member A (*RHOA)*, Fibroblast Growth Factor Receptor 3 (*FGFR3)*, Telomerase Reverse Transcriptase (*TERT)*, *Fms Related Receptor Tyrosine Kinase 4 (FLT4)*, Polymerase (DNA) Epsilon, Catalytic Subunit (*POLE)*, AKT serine/threonine kinase 1 (*AKT1)*, and Core-Binding Factor, Runt Domain, Alpha Subunit 2; Translocated To, 3 (*CBFA2T3)* were observed in the MPA cohort, as deletions of Metastasis Associated Lung Adenocarcinoma Transcript 1 (*MALAT1), RecQ Like Helicase 4 (RECQL4), Notch Receptor 1 (NOTCH1), Axis Inhibition Protein 1 (AXIN1), Calreticulin (CALR), Lymphoblastic Leukemia-Derived Sequence 1 (LYL1), Roundabout Guidance Receptor 2 (ROBO2), Caspase Recruitment Domain Family Member 11 (CARD11), Nuclear Receptor Corepressor 2 (NCOR2), AT-Rich Interaction Domain 1A (ARID1A), Mechanistic Target Of Rapamycin Kinase (MTOR), Ribosomal Protein L22 (RPL22), Sloan-Kettering Institute Proto-Oncogene (SKI), Tumor Necrosis Factor Receptor Superfamily, Member 14 (TNFRSF14), Calmodulin Binding Transcription Activator 1 (CAMTA1), PR Domain Containing 16 (PRDM16), Fibroblast Growth Factor Receptor 3 (FGFR3), RHOA*, and *AKT1*. The top driver genes located in these amplified or deleted regions were delineated by Venn diagrams (**Figure 2C**). *FLT4* amplification occurred in both MPA and LUAD cohorts, while *AKT1* was amplified in the MPA cohort but deleted in the LUAD cohort. We further explored the impact of the driver genes on OS contained within these copy number regions in MPA. Mutation of *AKT1* (*P*=1.00E-03) and *NKX2-1* (*P*=1.90E-02) was associated with significantly worse OS, suggesting the prognostic effect of *AKT1* and *NKX2-1* in the prediction of poor survival (**Figure 2D**). In MPA and LUAD cohorts, shared driver genes in CNV gain were found in *AKT1, B-Cell Lymphoma 9 Protein (BCL9)*, and *NKX2-1*, whereas CNV loss happened in *LYL1, Metastasis Associated Lung Adenocarcinoma Transcript 1 (MALAT1), Notch Receptor 1 (NOTCH1)*, and *AKT1*, with *AKT1* (47.92%) and *BCL9* (45.83%) showed the highest mutation frequency. Interestingly, the gain or loss of CNV was exclusive for MPA and LUAD cohorts (**Figure 2E**).

### Genome instability comparison

**Figure 3:**
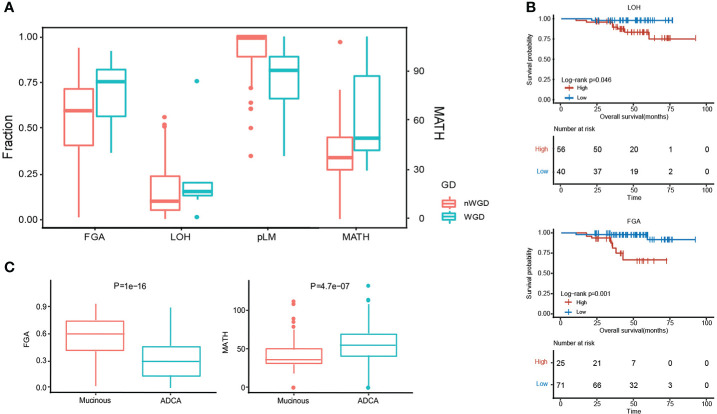
Genome instability comparison between MPA and LUAD. **(A)**, Evaluation of five genomic instability indexes in MPA. **(B)**, Survival analysis combing genomic instability indicators. **(C)**, Comparison of FGA and MATH between MPA and LUAD.

MPA tumorigenesis was correlated with genome instability, which has been also positively associated with the occurrence and development of carcinoma. We evaluated some genome features to evaluate the genome instability status in each patient using whether whole genome duplication (WGD), including the fraction of the genome altered (FGA), loss of heterozygosity (LOH), percentage of late mutations (pLM), and mutant-allele tumor heterogeneity (MATH). We showed the existence of WGD in patients with significantly higher FGA, LOH, and MATH, whereas a lower level of pLM (**Figure 3A**). Higher LOH (*P* = 4.60E-02) and FGA (*P*= 1.00E-03) expressions predicted worse survival (**Figure 3B**). MPA had significantly higher FGA (*P*=1.00E-16) and lower MATH (*P*=4.70E-07) in comparison with LUAD (**Figure 3C**).

### Mutational signatures

**Figure 4:**
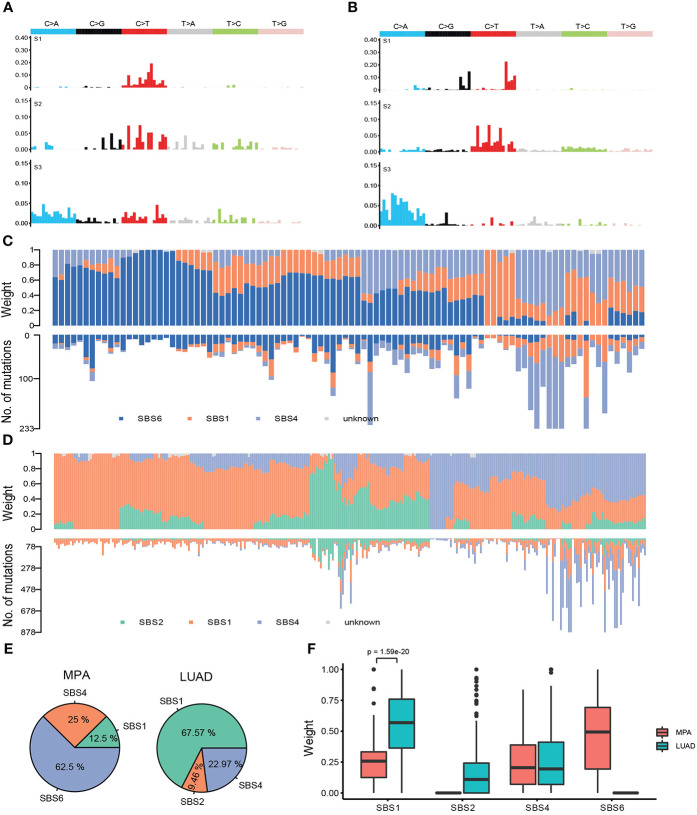
Mutational signatures of MPA and LUAD cohort. **(A)**, Single nucleotide variation analysis of the three signatures predicted in MPA. **(B)**, Single nucleotide variation analysis of the three signatures predicted in LUAD. **(C)**, The number of mutations in each sample of the three signatures predicted by MPA (lower part) and the proportion of the three signatures in each sample (upper part). **(D)**, The number of mutations in each sample of the three signatures predicted by LUAD (lower part) and the proportion of the three signatures in each sample (upper part). **(E)**, Proportion of samples for each signature in MPA and LUAD. **(F)**, The weight means of each signature in each sample. The *P*-value was obtained by the Wilcox test.

We further analyzed the mutational signatures of the MPA cohort, which identified four independent mutational signatures that matched four of the Sanger signatures in the COSMIC database, including SBS6, SBS1, SBS4, and an unknown feature (**Figure 4A**). The type of base-pair substitutions of each sample and mutational spectrum is demonstrated (**Figure 4A**). Mutation spectrum analysis revealed that the most common somatic substitutions were C-T transversions in MPAs and LUADs (**Figures 4A, B**), which was consistent with previous studies for LUAD (29). Other most common somatic substitutions in MPAs were C-A, C-G, T-C, T-A, and T-G. Three remarkable signature groups were clustered using the relative contributions of these substitutions. The number of mutations in each sample of the three signatures predicted by MPA and the proportion of the three signatures in each sample were shown in **Figure 4C**. Similarly, SBS2, SBS1, SBS4, and an unknown signature matched four of the Sanger signatures in the Catalogue of Somatic Mutations in Cancer (COSMIC) database (**Figure 4B**). The number of mutations in each sample of the three signatures predicted by LUAD and the proportion of the three signatures in each sample were shown in **Figure 4D**. The distribution of signatures revealed that 62.5% of all 96 cases had SBS6, which was the most common signature as expected. SBS4 was the second most common, accounting for 25% whereas SBS1 only accounted for 12.5% of all cases in our MPA cohort. The distribution of signatures in LUAD was found to be distinct from MPA in general, with SBS1 (67.57%) the most commonly detected in LUAD, followed by SBS4 (22.97%) and SBS2 (9.46%) (**Figure 4E**). The MPA and LUAD cohorts demonstrated significantly different signatures except for SBS4 (**Figure 4F**). Overall, the mutational signature suggest that MPAs had marked different genomic alterations and were more complex in genomic profiles than LUADs. These results indicated that different mutagenic processes related to DNA mismatch repair may be involved in the tumor genesis of MPA.

### Comparison of the alteration frequency of genes in pathways across MPA and LUAD

**Figure 5:**
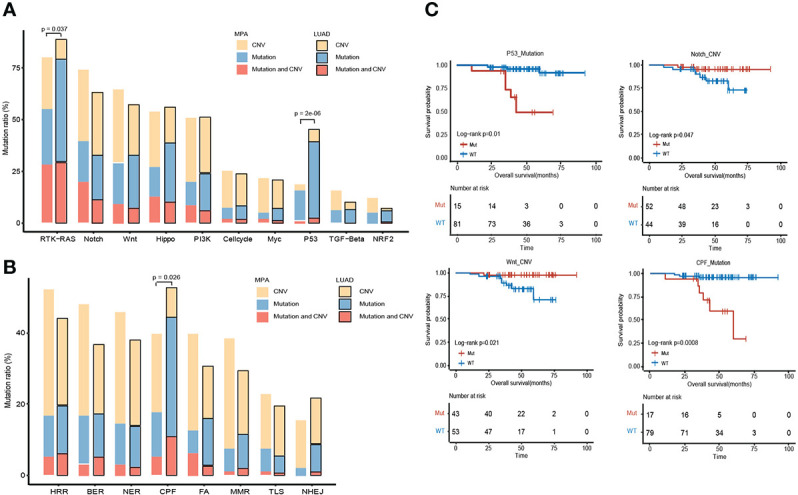
Comparison of the alteration frequency of genes in pathways across MPA and LUAD. **(A)**, Comparison of the alteration frequency of genes in ten hallmark pathways across the two cohorts. The yellow part point to the proportion of CNV only, the blue part is the proportion of only mutation, and the red part is the proportion of samples where CNV and mutation occur. The *p*-value marked on the column represents a fisher test result of samples with and without mutations in MPA and LUAD in this pathway. **(B)**, Comparison of the enrichment of mutant genes in 8 DDR pathways in MPA and LUAD. The yellow part indicates that only one proportion of CNV occurs, the blue part is the proportion of only mutations, and the red part is the proportion of samples that have both CNV and mutation. The *p*-value marked on the column represents a fisher test result of samples with and without mutations in MPA and LUAD in this pathway. **(C)**, Survival analysis results between mutant and wild type in 18 pathways. Only show the results of pathways with significant differences, and perform different analyses on CNV and mutation results.

The altered key pathways affected by CNV, somatic mutations, and integrated information were performed to construct a comprehensive view of aberrant characteristics for MPAs. Ten frequently altered hallmark pathways were shown in **Figure 5A**. Receptor Tyrosine Kinase (RTK)-RAS pathway was the most altered pathway in MPA and LUAD cohort, affecting 80.15% and 88.85% of their respective tumor mutations. The altered key pathways enriched in 8 DDR for MPA and LUAD were shown in **Figure 5B**. The most frequently altered pathways in MPA were HRR, affecting 52.09% of MPA mutations. Specifically, all 18 mutation pathways between mutant and wild type were analyzed in combination with survival information. In SNV mutation enrichment, mutations in P53 (*P* =1.00E-02) and CPF (*P*=8.00E-04) pathways significantly shortened survival (**Figure 5C**). At the CNV level mutation, Notch (*P* =4.70E-02) and Wnt (*P*=2.10E-02) pathways significantly prolonged survival (**Figure 5C**). Additionally, the frequency of both SNVs and CNV on the RTK-RAS, Notch, Wnt, Hippo, PI3K, P53, Cell cycle, Transforming Growth Factor Beta (TGF-beta), Myc, and Nuclear Factor, Erythroid 2 Like 2 (NRF2) of the oncogenic signaling pathways were demonstrated in MPA (**Figures S2A, B**). Similarly, enrichment of SNV and CNV mutations in 8 DDR pathways in MPA was also demonstrated (**Figures S2C, D**).

### Clinical analysis of immune-related mutant genes

**Figure 6:**
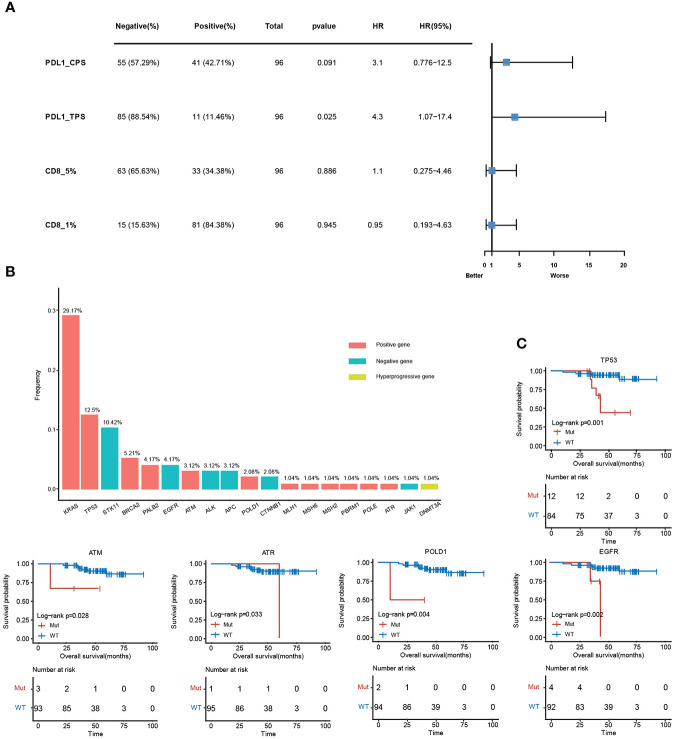
Clinical analysis of immune-related mutant genes. **(A)**, Survival analysis between positive and negative samples in the four immune indicators, PDL1-CPS, PDL1-TPS, CD8 (threshold is 5%), CD8 (threshold is 1%). **(B)**, The mutation frequency of immune-related genes. The red histograms are immune positively related genes, blue are immune negatively related genes, and yellow are immune hyper-progressive genes. **(C)**, Survival analysis results between mutant and wild-type immune-related genes.

The relative level of immune infiltration in tumors can be reflective of the immune microenvironment induced by a gene mutation. Expression profiles of immune-related mutant genes were examined for differently expressed genes. The survival was analyzed in samples with different immune status as evaluated by the four immune-related indicators (PDL1-CPS, PDL1-TPS, CD8-5%, CD8-1%). PDL1-TPS was associated with worse survival (*P* = 2.50E-02, HR = 4.3, 95% CI: 1.07-17.4) (**Figure 6A**). No significant association was found between PDL1-CPS and survival. Neither was found between CD8+ 5%, CD8+ 1% and survival. Statistical distribution of high-frequency somatic mutated genes in immune correlation samples were shown in **Figure 6B**. *KRAS* (29.17%) occupies the largest proportion of mutated genes in immune-positive correlated mutations, while *STK11* (10.42%) belongs to the immune-negative gene. DNA Methyltransferase 3 Alpha (*DNMT3A)* showed as the immune hyper-progressive genes (**Figure 6B**). Survival analysis between mutant and wild-type immune-related genes was conducted, and five of these genes showed significant differences among the subgroups. Mutations in *TP53, ATM, POLD1*, and *EGFR* were all correlated with worsened survival (*P <*0.05) (**Figure 6C**).

### Druggable genes

Among the mutant genes found in MPA samples, 42 have been depicted in the OncoKB database as targetable by known drugs (*ALK*, 2 drugs; *ATM*, 1 drug; *BRAF*, 10 drugs; Breast Cancer Type 2 Susceptibility Protein (*BRAC2)*, 5 drugs; Cyclin Dependent Kinase Inhibitor 2A (*CDKN2A)*, 3 drugs; *EGFR*, 7 drugs; *KRAS*, 5 drugs; RAD51 Homolog B (*RAD51B)*, 1 drug). The interaction between drug and gene is depicted in **Table 3**. For instance, in the study led by Planchard, Dabrafenib plus trametinib represents a new therapy with clinically meaningful antitumor activity and a manageable safety profile in patients with previously untreated BRAFV600E-mutant NSCLC. Clinical trials have been conducted among NSCLC patients harboring distinct mutations. In a clinical trial (NCT00525148), afatinib demonstrated activity in the treatment of patients with advanced lung adenocarcinoma with EGFR mutations, especially in patients harboring deletion 19 or L858R mutations. Notably, for *KRAS* p.G12C-mutatant patients with NSCLC, anticancer effect of sotorasib was detected. In its phase 2 trial (NCT03600883), sotorasib resulted in a durable benefit without new safety signals in patients with previously treated *KRAS* p.G12C-mutatant NSCLC. These genes have provided us clues on the possible drugs and distinct convincement that could be tapped for treatments.

**Table 3:** The targetable mutations and possible drugs in MPA.

Gene	HGVSp_Short	Number_sample	Drug	HIGHEST_LEVEL
ALK	p.S691_H694delinsMGPA	1	Lorlatinib,Brigatinib	LEVEL_1
ATM	p.D2708N	1	Olaparib	LEVEL_3B
ATM	p.R337L	1	Olaparib	LEVEL_3B
ATM	p.T2771Cfs*5	1	Olaparib	LEVEL_3B
BRAF	p.V600E	1	Dabrafenib+Trametinib;Dabrafenib,Encorafenib+Cetuximab,Selumetinib,Encorafenib+Panitumumab,Trametinib,Vemurafenib,Vemuraf	LEVEL_1
BRCA2	p.L1635Ffs*15	1	Olaparib,Talazoparib,Olaparib+Bevacizumab,Rucaparib,Niraparib	LEVEL_3B
BRCA2	p.S744*	1	Olaparib,Talazoparib,Olaparib+Bevacizumab,Rucaparib,Niraparib	LEVEL_3B
CDKN2A	p.A76Sfs*85	1	Palbociclib,Ribociclib,Abemaciclib	LEVEL_4
CDKN2A	p.G69Wfs*104	1	Palbociclib,Ribociclib,Abemaciclib	LEVEL_4
CDKN2A	p.Y44Tfs*85	1	Palbociclib,Ribociclib,Abemaciclib	LEVEL_4
EGFR	p.G719S	1	Afatinib	LEVEL_1
EGFR	p.L858R	1	Erlotinib,Erlotinib+Ramucirumab,Afatinib,Gefitinib,Osimertinib,Dacomitinib	LEVEL_1
EGFR	p.S768I	1	Afatinib	LEVEL_1
KRAS	p.G12A	1	Trametinib,Cobimetinib,Binimetinib	LEVEL_4
KRAS	p.G12C	3	Sotorasib;Adagrasib;Trametinib,Cobimetinib,Binimetinib	LEVEL_1
KRAS	p.G12D	8	Trametinib,Cobimetinib,Binimetinib	LEVEL_4
KRAS	p.G12V	15	Trametinib,Cobimetinib,Binimetinib	LEVEL_4
KRAS	p. Q61H	1	Trametinib,Cobimetinib,Binimetinib	LEVEL_4
RAD51B	p.Q371*	1	Olaparib	LEVEL_3B

## Discussion

This study offers us a novel insight into MPA, a rare cancer with limited therapeutic options, by tapping deeper into its biological features and making a comparison with LUAD. We aimed to explore novel therapeutic strategies for this rare and lethal cancer by WES, which represents the largest Chinese MPA cohort. We depicted a comprehensive genomic and immunological landscape of this rare cancer and identified several genomic and immune-related features that were associated with clinical outcomes.

MPA has long been considered a tricky malignancy due to the elusiveness of its genomic nature (30). Our study revealed that *KRAS* was the most frequent mutation gene in MPA. Similarly, *KRAS* was reported to be the most frequent mutation in other studies despite its mutation frequency varies. In the previous study (9), we found 63% MPA cases harbored *KRAS* mutation, while it showed a far high level of 29.17% in our cohort. *TP53*, a key tumor suppressor that plays a major role in preserving genomic stability (31), was found to be mutated at a frequency of 12.50% in our study, whereas only 2 out of the 50 cases were identified to be *TP53*-mutant in a previous study (9). The discrepancies in these genetic mutation frequencies might be due to the differences in the sequencing platform and study population. Given the low incidence of MPA, collaborative efforts across different nations are desperately needed for a large-scale genomic analysis in diverse populations to delineate the genomic landscape of this rare disease.

Notably, we also revealed the mutations of *NKX2-1* and *ERBB2* in our Chinese MPA cohort, which could be hardly detected in the LUAD counterpart. Moreover, transgenic mice with inactivated *NKX2-1/Ttf-1* were demonstrated to develop MPA in the lungs that resemble human MPA (32). Besides, *ERBB2* reported to be mutated in never smokers among LUAD patients previously (33), was found to be mutated in the MPA cohort. The interest in the identification of *ERBB2* mutation in MPA is driven by recent therapeutic strategies involving anti-Human Epidermal Growth Factor Receptor 2 (anti-*HER2)* therapy that have demonstrated durable responses in patients with advanced tumors harboring these mutations. For instance, the DESTINY-Lung01 trial targeting *ERBB2* alterations indicates promising clinical effects of anti-*HER2* therapy (34). In summary, the present study has indicated the potential role of these mutant genes in MPA development. And the concrete role of these mutations in the tumorigenesis and tumor progression in MPA warrants our further exploration.

For further comparison of the somatic mutation of MPA with those of LUAD, we have also depicted the disparity between them, which may reveal their distinct clinicopathological and biological features. A significant difference was found in age and clinical stage between MPA and LUAD. Many cancers are age-associated and aging is associated with loss of function in many tissues. The progressive accumulation of mutations in oncogenes and tumor suppressors contribute to the oncogenesis (35, 36). Additionally, genomic alterations are acquired during the evolution of cancers from their early to late stages. Thus, tumors at various stages may harbor different genetic mutations. Therefore, it is possible that age distribution and stage distribution disparities may influence the genetic landscape of MPA and LUAD. In particular, CUB And Sushi Multiple Domains 1 (*CSMD1)* has caught our attention since it has been reported to be mutated in a variety of cancers. As a regulator of complement activation and inflammation, it was considered a tumor suppressor in advanced oral, gastric, prostate, and breast cancer (37–39). Loss of its functionality is linked with poor prognosis and enhanced proliferation, migration, and invasion (40, 41). Concerning CNV, LUAD had significantly more copy number amplification and deletion than MPA. Therefore, we can readily distinguish the two cohorts according to the somatic mutation and copy number profiles.

For the mutational signature analysis, we found that MPAs were predominantly associated with signature 1, signature 4 (smoking), and signature 6 (defective DNA mismatch repair). These findings suggest that MPA is similar to conventional LUAD which is intimately associated with smoking signatures. However, owing to the rarity or absence of targetable mutations, the targetable treatments were difficult to be explored. Several studies have indicated that *KRAS* mutations could be targeted for therapeutic intervention, however, its efficacy for MPA patients has not been confirmed in clinical practice yet.

Genomic instability has been considered a hallmark of cancers with unfavorable outcomes. In the present study, we demonstrated genomic instability in MPA, featured by FGA, LOH, pLM, and MATH, highlighting the dismal nature of MPA and the therapeutic challenges we are faced with. Notably, TMB was significantly higher in LUAD than that of MPA. Interestingly, no difference in smoking status was found between the two cohorts. We, therefore, speculated that the significantly higher TMB in LUAD was not due to the chronic DNA damage resulting from smoking. Rather, the *TP53* status may be the dominant contributor to the disparity in mutational burden. Besides, both intratumor genetic heterogeneity and tumor purity can affect the TMB. Thus, these are also two important factors that should be taken into account and remain in our further exploration.

Furthermore, concerning developing immune checkpoint inhibitor treatment in the context of mutant genes, it would be vital to consider the therapeutic indications of mutation frequency of immune-related genes. Mutation of *KRAS* tops other genes that were possibly associated with improved immunotherapy responses, followed by *p53*. It has been documented that *KRAS* mutations can enhance PD-L1 expression, promote T cell infiltration and enhance tumor immunogenicity (42). This is attributed to the association between smoking and the presence of *KRAS* mutation. LUAD harboring *TP53* mutation had elevated PD-L1 and a high somatic mutation burden, which may account for its increased immune response. Clinical trials have confirmed the significant clinical benefit for patients with *KRAS* mutation to receive PD-1 inhibitors among NSCLC. However, the beneficial role of *KRAS* mutation in response to immunotherapy among the MPA cohort warrants our further exploration.

The detection of genetic profiling could be helpful in the selection of possible effective drugs in MPA. The *KRAS* mutation rate in MPA has been reported to be up to 60%, with the most ubiquitous variant of G12D and G12V (9). Besides, *KRAS* G12C has also been found to be a potentially targetable variant. Notably, for *KRAS* p.G12C-mutated patients with NSCLC, sotorasib has demonstrated its promising anticancer activity. In its phase 2 trial (NCT03600883), treatment with sotorasib led to a durable clinical benefit in patients with previously treated *KRAS* p.G12C-mutated NSCLC (43).

Undeniably, our present study has several limitations. First, we did not distinguish between pure MPA and mixed MPA in detail. Second is the possibility that low tumor purity is an issue with the sequencing of MPA. There are possibilities that low tumor purity might have affected the genomic results in some cases. Third, MPA could be further categorized into different classifications such as primary signet-ring cell carcinoma, primary mucinous bronchioloalveolar carcinoma, primary mucinous colloid adenocarcinoma, et al. These distinct classifications may exhibit their unique features in pathology, genetic alteration and survival. It is a pity that we do not further divide MPA into these classifications further. However, several strengths of our study are noteworthy. First, we provided the comprehensive landscape of SNV, CNV, genomic instability, involved pathways, and immune-related features associated with survival in our Chinese MPA. All of the prior studies were focused on either SNV or CNV features of MPA. Second, we compared many of the genomic and immunological features with LUAD, which could suggest distinct therapeutic targets and guide clinical management in MPA, as distinct from LUAD. Third, it has to be noted that most of the patients in our study were not subject to extensive therapies, therefore the genomic profiles should not have been affected by the treatments.

In conclusion, we conducted the largest WES encompassing CNV, SNV, genomic instability, and immunological features of Chinese MPA patients to date. The study boosts our understanding of the complex molecular constituent of MPA and reveals that the underlying genomic alterations could be exploited for better distinction with LUAD and new treatment options. Despite a relatively smaller number, our study provides us an overview of the genetic landscape of the rare disease, which may offer a rationale for targeted therapeutic strategies for MPA.

## Data availability statement

The datasets presented in this study can be found in online repositories. The names of the repository/repositories and accession number(s) can be found below: Raw sequencing data were deposited in the Genome Sequencing Archive (GSA) of the China National Center for Bioinformation (CNCB) (https://ngdc.cncb.ac.cn/gsa/) under accession number PRJCA010644.

## Ethics statement

The studies involving human participants were reviewed and approved by Shandong Cancer Hospital and Institute. The patients/participants provided their written informed consent to participate in this study.

## Author contributions

CYZ, KW, and HaW contributed to the conception and design of the work. Collection of data was conducted by JL, ZL and HuW. Analysis and interpretation of the data were performed by WL, CLZ, YC, SW, AY, and JW. Drafting of the manuscript was done by CYZ and KW. Editing of the work was done by CYZ and JL. Final approval of the work was done by all authors. CYZ, KW, WL, and JL contributed equally to this work and co-first authors. All authors contributed to the article and approved the submitted version.
