# Supplementary figures and images for: Whole exome sequencing in Chinese mucinous pulmonary adenocarcinoma uncovers specific genetic variations different from lung adenocarcinoma
Source: Front Oncol. 2022 Dec 15;12:1054845. doi: 10.3389/fonc.2022.1054845 (PMC9798319; doi:10.3389/fonc.2022.1054845)

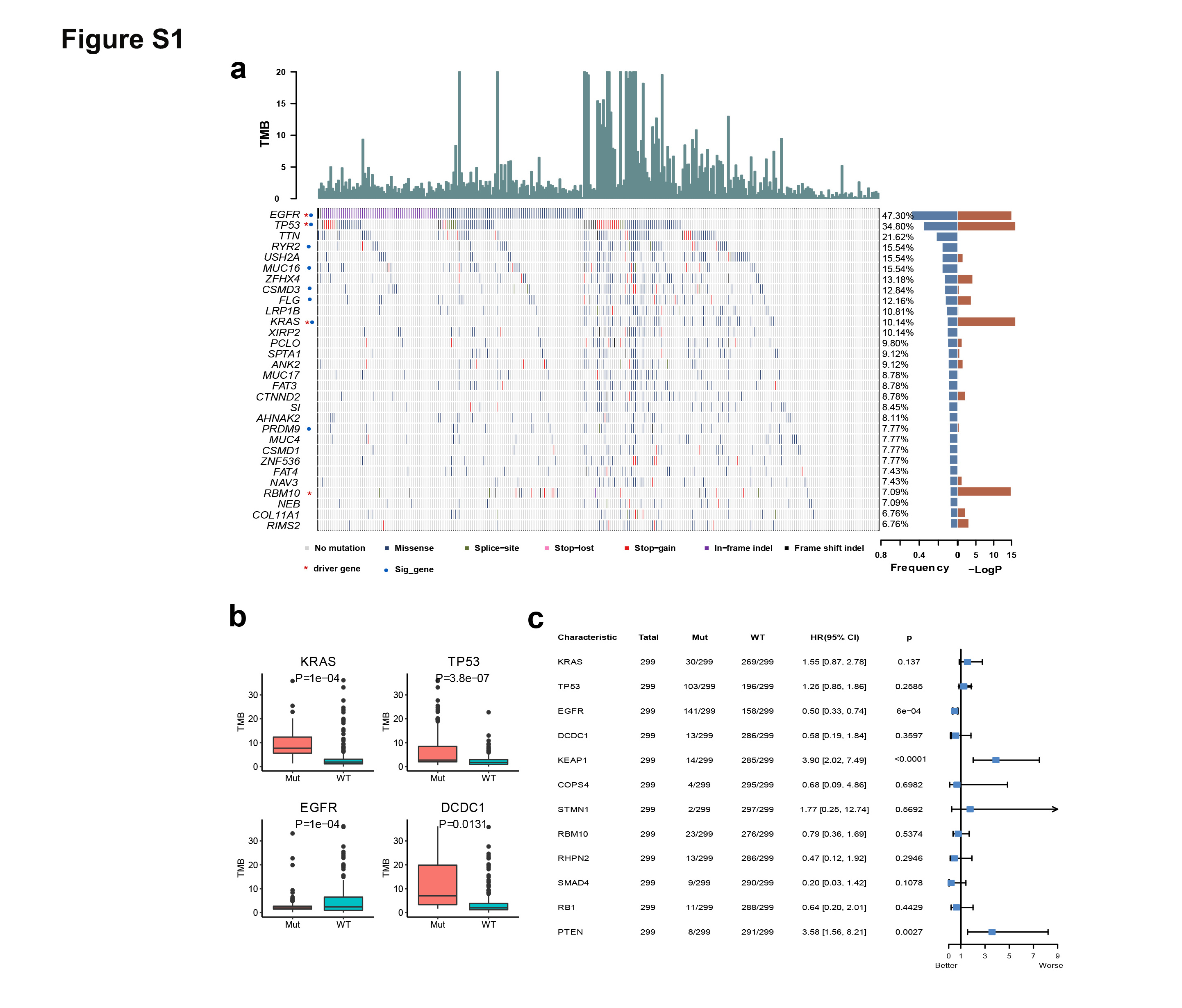

Supplement: Supplementary file 1 [file Image_1.jpeg]

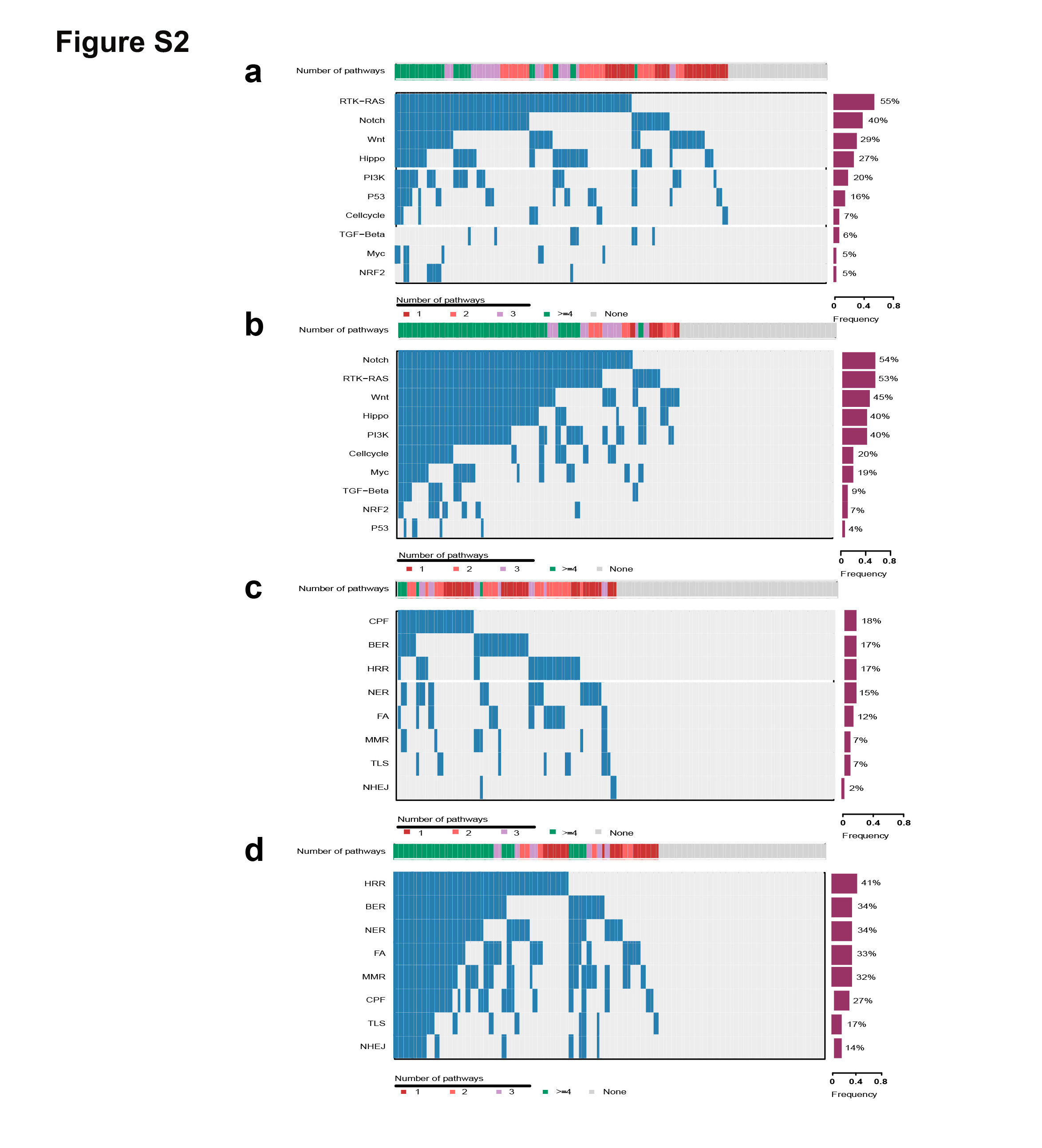

Supplement: Supplementary file 2 [file Image_2.jpeg]
