# Supplementary material for: Whole exome sequencing in Chinese mucinous pulmonary adenocarcinoma uncovers specific genetic variations different from lung adenocarcinoma
Source: Front Oncol. 2022 Dec 15;12:1054845. doi: 10.3389/fonc.2022.1054845 (PMC9798319; doi:10.3389/fonc.2022.1054845)
Supplement: Supplementary file 3 [file DataSheet_1.doc]

**Supplementary figure legends**

**Figure S1 Somatic mutations and clinical association in LUAD**

**a**, Spectrogram of top30 mutant gene in LUAD. The blue in the histogram on the right represents the mutation frequency of the gene, and the red is the -logP value when predicting whether the gene is a driving gene. There are red asterisks (driver genes predicted by the software) and blue circles (genes with significant difference in mutation frequency between lung mucinous adenocarcinoma and lung adenocarcinoma) on the right of the gene name on the left. **b**, Comparison of TMB between mutant and wild-type driver genes in LUAD. **c**, Survival analysis results of LUAD driver genes.

**Figure S2 Enrichment of SNV and CNV mutations in 10 hallmark and 8 DDR pathways in MPA**

**a**, Enrichment of SNV mutations in 10 hallmark pathways in MPA. **b**, Enrichment of CNV mutations in 10 hallmark pathways in MPA. **c**, Enrichment of SNV mutations in 8 DDR pathways in MPA. **d**, Enrichment of CNV mutations in 8 DDR pathways in MPA.
