# Supplementary material for: Whole exome sequencing in Chinese mucinous pulmonary adenocarcinoma uncovers specific genetic variations different from lung adenocarcinoma
Source: Front Oncol. 2022 Dec 15;12:1054845. doi: 10.3389/fonc.2022.1054845 (PMC9798319; doi:10.3389/fonc.2022.1054845)
Supplement: Supplementary file 4 [file DataSheet_2.doc]

**Supplementary materials and methods**

**Tumor heterogeneity and genome instability analysis**

To investigate intratumor heterogeneity (ITH), mutant allele tumor heterogeneity (MATH) values for each tumor sample were calculated from the median absolute deviation (MAD) and the median of its mutant-allele fractions at tumor-specific mutated loci: MATH = 100 × MAD/median. These analyses were performed in R with default parameters as previously reported [1]. Cancer cell fraction (CCF), and clonal and subclonal mutations in each tumor specimen were calculated based on the proportion of mutated reads (variant allele fraction, VAF) as previously reported [2]. Regarding genome instability analyses, cellular purity, ploidy, and segmented allele-specific copy number profiles of each specimen tumor cell were estimated using Sequenza [3]. The fraction of genome altered (FGA) was defined as the percentage of a tumor genome harboring copy number variations against the whole genome. Loss-of-heterozygosity (LOH) segments or mutations were defined by the minor allele copy number or mutation ratio < 0.25 [4]. Whole-genome doubling (WGD) events were defined as the major allele ploidy >1.5 on at least 70% of at least 11 autosomes as the duplicated autosomes number per sample [5].

**The evaluation of PD-L1 expression**

For the evaluation of PD-L1 expression, TPS was defined as the number of PD-L1-staining tumor cells divided by the total number of viable tumor cells multiplied by 100. CPS was defined as the number of PD-L1-staining cells divided by the total number of viable tumor cells multiplied by 100 [6]. PD-L1 staining of tonsil has been adopted to ensure eligibility of the enrolled specimens. The qualified staining should be: strong positivity for PD-L1 in intratonsillar cleft epithelium whereas negative staining for PD-L1 in lymphocytes (mantle zone and germinal center B cells) and superficial epithelial cells.

**The calculation of CD8+ T cell infiltration**

We observe whether CD8+ T cells were distributed uniformly in the tumor stroma at lower magnification. If they were equally distributed, they were calculated in three randomly-chosen areas (0.1mm^2^ per area) at a 200-fold magnification. If unequally distributed, corresponding areas would be selected at a 200-fold magnification according to the percentage of CD8^+^ T cell in areas of various densities (0.1mm^2^ per area), as referred from the evaluation criteria for PD-L1 expression. The calculation is defined as: the count of CD8^+^ T cell/0.1 mm^2^×10, namely CD8+ T cell count/mm^2^. The cutoff values for CD8+ T cell infiltration were set at 1% and 5% respectively.

**References:**

1. Mroz EA, Rocco JW. MATH, a novel measure of intratumor genetic heterogeneity, is high in poor-outcome classes of head and neck squamous cell carcinoma. Oral Oncol. 2013;49(3):211-5.

2. Letouzé E, Shinde J, Renault V, Couchy G, Blanc JF, Tubacher E, et al. Mutational signatures reveal the dynamic interplay of risk factors and cellular processes during liver tumorigenesis. Nat Commun. 2017;8(1):1315.

3. Favero F, Joshi T, Marquard AM, Birkbak NJ, Krzystanek M, Li Q, et al. Sequenza: allele-specific copy number and mutation profiles from tumor sequencing data. Ann Oncol. 2015;26(1):64-70.

4. López S, Lim EL, Horswell S, Haase K, Huebner A, Dietzen M, et al. Interplay between whole-genome doubling and the accumulation of deleterious alterations in cancer evolution. Nat Genet. 2020;52(3):283-293.

5. Priestley P, Baber J, Lolkema MP, Steeghs N, de Bruijn E, Shale C, et al. Pan-cancer whole-genome analyses of metastatic solid tumours. Nature. 2019;575(7781):210-216.

6. Doroshow DB, Bhalla S, Beasley MB, Sholl LM, Kerr KM, Gnjatic S, Wistuba II, Rimm DL, Tsao MS, Hirsch FR. PD-L1 as a biomarker of response to immune-checkpoint inhibitors. Nat Rev Clin Oncol. 2021;18(6):345-362.
